# Polymorphisms of *SORBS*1 Gene and Their Correlation with Milk Fat Traits of Cattleyak

**DOI:** 10.3390/ani11123461

**Published:** 2021-12-05

**Authors:** Luyu Yang, Xingyu Min, Yanjin Zhu, Yulei Hu, Manzhen Yang, Hailing Yu, Jian Li, Xianrong Xiong

**Affiliations:** 1 Key Laboratory of Qinghai-Tibetan Plateau Animal Genetic Reservation and Exploitation of Ministry of Education, Southwest Minzu University, Chengdu 610041, China; yangluyu0413@yeah.net (L.Y.); minxingyu2333@163.com (X.M.); zhuyj199434@163.com (Y.Z.); stonetear@163.com (Y.H.); jianli_1967@163.com (J.L.); 2 Key Laboratory of Animal Science of National Ethnic Affairs Commission, Southwest Minzu University, Chengdu 610041, China; yangmanzhen2021@163.com (M.Y.); HaiLingYu96@163.com (H.Y.)

**Keywords:** *SORBS*1 gene, cattleyak, milk fat traits, SNPs, diplotype

## Abstract

**Simple Summary:**

Increasing milk fat rate has a good effect on the milk quality of cattleyak. SNPs can help us find potential molecular markers for the milk fat traits of cattleyak, and they can be screened according to molecular markers when they are young. It provides a reference for cultivating high milk fat cattle population in the future. The results of this study suggest that the *SORBS*1 gene polymorphism is closely related to the milk fat traits of cattleyak, which could be used as a candidate genetic marker for milk fat trait selection in cattleyak. This study provides a new molecular marker and theoretical basis for screening the milk fat traits of cattleyak. It has a certain reference value for the research and improvement of milk quality.

**Abstract:**

This study aimed to find the SNPs in the *SORBS*1 gene of cattleyak, analyze the relationship between its polymorphisms and the milk fat traits, and find potential molecular markers for the milk fat traits of cattleyak. The polymorphism of the *SORBS*1 gene in 350 cattleyak from Hongyuan County (Sichuan, China) were detected by PCR and DNA sequencing, and the correlation between these SNPs and the milk production traits of cattleyak was analyzed. The results showed that there were nine SNPs in the CDS and their adjacent non-coding regions of the *SORBS*1 gene, and all SNPs have three genotypes. The correlation analysis found that the genotypes with superior milk fat traits in the other eight alleles were homozygous genotypes with a high genotype frequency except the g.96284 G > A (c.3090 G > A) (*p* < 0.05). However, at locus g.96284 G > A, the milk fat percentage, monounsaturated fatty acids (MUFAs), polyunsaturated fatty acids (PUFAs) and saturated fatty acids (SFAs) of the GA genotype were significantly higher than that of GG and AA genotypes (*p* < 0.05). Among these SNPs, three SNPs (g.6256 C > T (c.298 C > T), g.24791 A > G (c.706 A > G) and g.29121 A > G (c.979 A > G)) caused the amino acids change. The genotypes of the three SNPs consist of three haplotypes and four diplotypes. The amino acid mutation degree of diplotype H1–H1 (CCAAAA) was the highest, and its milk fat percentage, MUFAs, PUFAs and SFAs were also the highest (*p* < 0.05). Taken together, we found nine SNPs in the *SORBS*1 gene that are closely related to the milk fat traits of cattleyak. Moreover, the mutation of amino acids caused by SNPs had positive effects on the milk fat traits of cattleyak. H1-H1 is the dominant diplotype which significantly related to the milk fat traits of cattleyak. This study provides a new molecular marker and theoretical basis for screening the milk fat traits of cattleyak.

## 1. Introduction

Yak (Bos grunniens), a wild bovine species that lives 3500 m above sea level, is an iconic symbol of the Qinghai–Tibetan Plateau region and the nearby areas. Moreover, yak raising has become the main livelihood in the local area. However, the reproductive efficiency and production performance of yak are low, which cannot satisfy the local herdsman needs and industrial development [1]. As the hybrid offspring of yak (♀) and cattle (Bos taurus) (♂), the female cattleyak has obvious heterosis compared to yak, especially in its lactation performance [2,3]. Dairy products made from fermented and processed milk are popular with local herdsmen and tourists. As the main energy substance in milk, milk fat only accounts for 3~5% of the milk content, but it determines the nutritional value of milk [4,5]. Therefore, it is greatly meaningful to increase the percentage of milk fat and improve the composition of fatty acids in cattleyak.

The relevance between polymorphism and milk quality has been widely applied to the breeding of lactation traits, and the relationship between milk composition and genetic variation in milk production has a far-reaching impact on milk production [6,7,8,9,10,11,12]. In genetics, milk fatty acids have been found to be heritable, and the heritability was estimated at 0.22 to 0.71 [13]. In the past few years, with the rapid development of biotechnology, the mapping of candidate genes and quantitative trait locus (QTL) has been gradually applied to analyze lactation traits and breeding. Therefore, some promising SNPs have been shown to be significantly associated with lactation and its milk components [14,15,16].

The sorbin and SH3 domain-containing 1 (*SORBS*1) gene is a member of the SORBS family, and it encodes a Cbl-associated protein (CAP) that plays key roles in the signaling and stimulation of insulin [17,18,19]. Previous studies found that after a postprandial lipid (PPL) challenge, fatty acids and sterols related to cholesterol absorption were increased, while sterols related to cholesterol synthesis were decreased, and they detected that two SNPs (rs.12247017 and rs.12240292) in the *SORBS*1 gene were correlated with b-Sitosterol after correcting for multiple testing [20]. In addition, *SORBS*1 blocks the insulin stimulation of glucose transport in 3T3-L1 adipocytes [21]. The T228A polymorphism of *SORBS*1 is correlated with obesity and diabetes [22]. Recently, it was found that the *SORBS*1 gene has an effect on cell proliferation and migration, which can inhibit tumor metastasis and improve the sensitivity of tumors to chemotherapeutic drugs [23]. Above all, *SORBS*1 has multiple functions, but there are still many potential mechanisms that need to be studied.

A genome-wide association study revealed that the *SORBS*1 gene was one of 20 new candidate genes that were closely related to milk fat traits in Chinese Holstein cows [24]. However, there are few studies on the breeding of the milk fat traits of cattleyak, and there is no report on the association between cattleyak and the *SORBS*1 gene. Therefore, whether the *SORBS*1 gene is related to the creamy traits of cattleyak remains to be determined. The cattleyak is the subject of this experiment, the detected SNPs of the *SORBS*1 gene and its association with cattleyak milk fat traits was analyzed, and we looked for the molecular markers for the assisted selection of the milk fat traits of cattleyak.

## 2. Materials and Methods

### 2.1. Animal and Preparation

The study samples and data were collected in Ngawa Tibetan and Qiang Autonomous Prefecture Hongyuan County, Sichuan Province, China (longitudinal 32°78′ N to 32°82′ N, latitudinal 102°52′ E to 102°55′ E). In total, 350 female cattleyak (Jersey cattle ♂ × yak ♀) were selected in this experiment. The female parent of our cattleyak comes from a group of relatively closed and natural mating yak, while the male parent comes from the frozen semen of the same batch of Jersey cattle. All animals were collected from August, and they have the same parity and lactation, a similar growth environment and equivalent nutritional conditions. The 25 mL milk samples of each cattleyak were collected and sent to the Hongya Yangping branch of New Hope Co., Ltd. The physical and chemical indexes (DHI) of each sample were analyzed by a milk composition analyzer. The 10 mL blood samples were collected from the jugular vein of each cattleyak by using a sterile blood collection device, and then put into the 4 °C ice box. All blood samples were taken back to the laboratory and placed in a freezer at −20 °C.

### 2.2. DNA Extraction from Blood

The DNA was extracted from whole-blood samples using the Blood Genomic DNA Extraction Kit (Solarbio, Beijing, China). The purity and concentration of the DNA were detected by a nucleic acid concentration detector, and the integrity of the DNA was then detected by agarose gel electrophoresis.

### 2.3. Primer Design and Sequencing

The standard sequence is from NCBI, which has 31 exons and 31 introns (Accession Number: NW_005394038.1). Based on the DNA sequence, 12 pairs of primers were designed for the PCR amplification of the *SORBS*1 gene CDS and their adjacent regions (Table 1). The PCR reaction was 25 µL: 12.5 µL 2× Rapid Taq Master Mix, l µL upstream and 1 µL downstream primers, 1 µL DNA template and add ddH2O to 25 µL. PCR reaction conditions were as follows: denaturation for 3 min at 95 °C, 35 cycles for 15 s at 95 °C, annealing temperature for 15 s and 72 °C for 15 s, and extension at 72 °C for 5 min. PCR products were detected by 2% agarose gel electrophoresis, and sent to Tsingke Biotechnology Co., Ltd. for sequencing.

### 2.4. Genotyping and Statistical Analysis

Based on the sequencing results, Chromas 2 software was used to analyze and find SNP sites. Using Excel 2019 to calculate the genotype frequency and allele frequency, the polymorphic information content (*PIC*) was calculated with different genotype frequency and allele frequency. The formula is as follows:

$$PIC=1-H_{0}-\sum_{i=1}^{n-1}\sum_{j-i+1}^{n}2Pi^{2}Pj^{2}$$

*Pi* and *Pj* are the frequencies of the *i* and *j* alleles, and *n* is the number of alleles. The Standard Amino acid sequence was derived from NCBI and compared with the pre- and post-mutation amino acid sequence in BLAST to test if the SNPs changed the amino acid sequence. Haploview 4.2 software was used to analyze the haplotype. The effect of different genotypes and diplotypes on the milk fat traits of cattleyak was analyzed by the Analyze–General linear model univariate in SPSS 19.0 software. The formula is as follows:

*Y*_*i*_ = *µ* + *G*_*i*_ + *e*

Among them, *Y_i_* is the phenotypic value of milk fat traits, and *µ* is the population mean of milk fat traits, *G_i_* is the total effect and *e* is the random residual effect. Differences were considered significant at *p* < 0.05.

## 3. Results

### 3.1. PCR Amplification and SNP Screening

**Figure 1:**
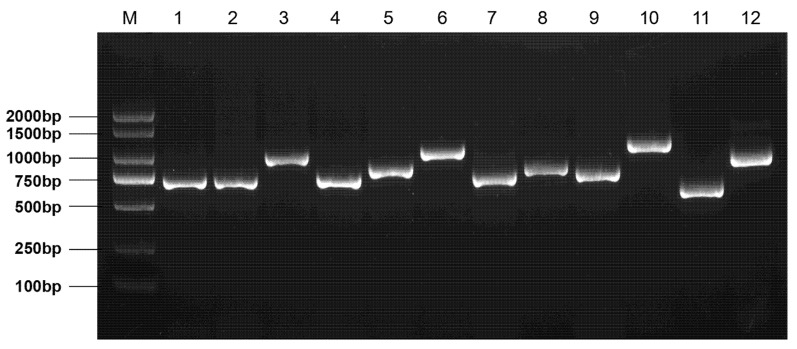
PCR amplification products (12 pairs of primers) for CDS and their adjacent regions of the *SORBS*1 gene.

The concentration and purity of genomic DNA in cattleyak blood were detected by a nucleic acid concentration detector, and the samples were all qualified. Using 2% agarose gel electrophoresis to detect four amplified products, clear and single-purpose bands were obtained. The specificity is good and can be used for subsequent sequencing analysis (Figure 1).

**Figure 2:**
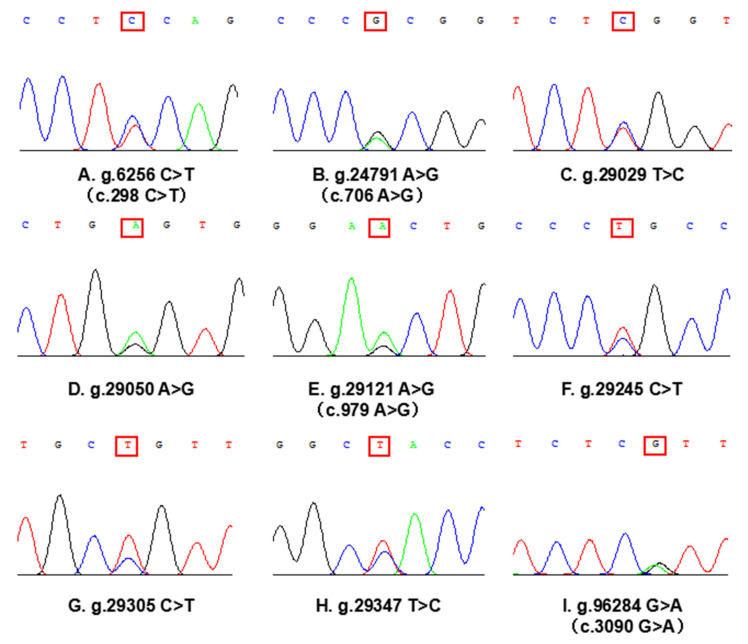
Sequencing diagram of heterozygous genotypes in the SNP loci of the *SORBS*1 gene (red box marker). Four SNPs (**A**,**B**,**E**,**I**) are located in the CDS region of the *SORBS*1 gene, c. system naming indicates the position of the site on the cDNA sequence. Five SNPs (**C**,**D**,**F**,**G**,**H**) are located in the intron region adjacent to the CDS region of the *SORBS*1 gene.

A total of nine SNPs in the CDS and their adjacent regions of the *SORBS*1 gene were detected in the cattleyak population by PCR and sequencing (Figure 2). g.6256 C > T (c.298 C > T) and g.24791 G > A (c.706 A > G) were detected by primer 1 and primer 4 PCR product, g.29029 C > T, g.29050 A > G, g.29121 A > G (c.979 A > G), g.29245 T > C, g.29305 T > C and g.29347 T > C were detected by primer 5 PCR product, g.96284 G > A (c.3090 G > A) was detected by primer 10 PCR product. Additionally, four SNPs (g.6256 C > T, g.24791 G > A, g.29121 A > G and g.96284 G > A) are located in the CDS region, while the other five SNPs are located in the intron region adjacent of the *SORBS*1 gene.

### 3.2. SNP Genotyping of the SORBS1 Gene

The genotype frequency, allele frequency and polymorphic information content of the nine SNP loci were shown in Table 2. The results showed that all the nine SNP loci had three genotypes in the cattleyak population. The PIC of g.6256 C > T, g.24791 G > A, g.29029 C > T and g.29050 A > G were 0.361, g.29121 A > G was 0.346, g.29245 T > C, g.29305 T > C, g.29347 T > C and g.96284 G > A were 0.358. The PIC of all SNPs was between 0.25~0.5, and all of them were moderately polymorphic. All the other SNPs conformed to the Hardy–Weinberg balance except g.29121 A > G site (*p* > 0.05).

### 3.3. Haplotype and Diplotype Analysis of Each Amino Acid Mutation Site of SORBS1

**Figure 3:**
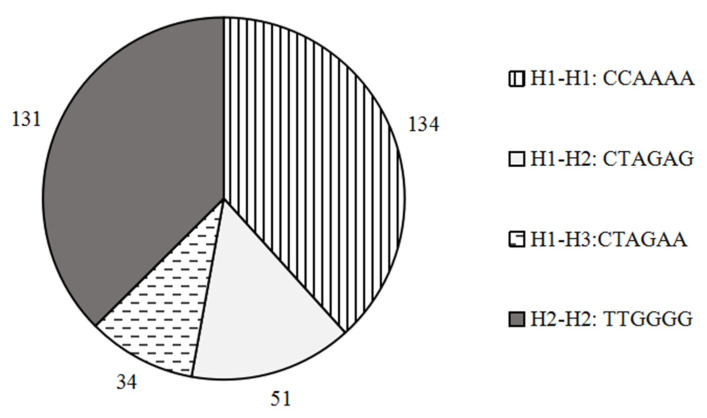
Diplotypes and frequencies of the three mutations in the *SORBS*1 gene.

During the comparison of the amino acid residue sequence alignment of the *SORBS*1 gene before and after mutation, we found that three SNPs (g.6256 C > T, g.24791 A > G and g.29121 A > G) caused amino acid changes among the four SNPs located in the CDS region. Moreover, no amino acid changes were observed at g.96284 G > A. The three mutations were Ser100Pro, Arg229His and Ala327Thr (Table 2). Using the three SNPs, a haplotype analysis was carried out using Haploview software (Table 3). The haplotype analysis showed that the three haplotypes were in fact found in the population of cattleyak, of which H1 (C-A-A) and H2 (T-G-G) were the major haplotypes, accounting for 61.9% and 33.3%, respectively. The frequency of H3 (T-G-A) was 4.9%. Based on these three haplotypes, we further obtained four combinations, in the form of H1–H1 (CCAAAA), H1–H2 (CTAGAG), H1–H3 (CTAGAA) and H2–H2 (TTGGGG) (Figure 3), of which H1–H1 (38.3%) and H2–H2 (37.4%) belonged to dominant diplotypes.

### 3.4. Association Analysis between SNP Genotypes and Milk Fat Traits

Based on the data of the SNP genotyping, the correlation analysis of a single locus with the milk fat percentage and fatty acids of cattleyak was carried out. As shown in Table 4, in terms of milk fat percentage and fatty acid content, most of the homozygous (CC genotype at g.6256 C > T, AA genotype at g.24791 A > G, TT genotype at g.29029 T > C, AA genotype at g.29050 A > G, AA genotype at g.29121 A > G, CC genotype at g.29245 C > T, CC genotype at g.29305 C > T, TT genotype at g.29347 T > C and GA genotype at g.96284 G > A) were significantly higher than the other genotypes (*p* < 0.05). At locus g.96284 G > A, the milk fat percentage, monounsaturated fatty acids (MUFAs), polyunsaturated fatty acids (PUFAs) and saturated fatty acids (SFAs) of the GA genotype were significantly higher than that in GG and AA genotypes (*p* < 0.05).

### 3.5. Association Analysis between Genotype Combinations and the Milk Fat Traits of Cattleyak

The genotype combination of H2–H2 did not cause the change of amino acid. There is a possibility of amino acid variation in the part of the cattleyak whose genotype combinations are H1–H2 and H1–H3. The base mutation in the combination of H1–H1 resulted in the mutation of all three amino acids. The results of the ANOVA analysis showed that H1–H1 had the highest milk fat percentage, MUFAs, PUFAs and SFAs, while H2–H2 had the lowest milk fat percentage and fatty acid content (Table 5). The differences were statistically significant (*p* < 0.05).

## 4. Discussion

Recently, the study of the polymorphism has been used to optimize the performance and composition of dairy cows [25,26,27,28]. The GWAS data in Li and his colleagues’ study suggested that a SNP in the *SORBS*1 gene can significantly influence the composition of fatty acids in milk, especially MUFAs and PUFAs [24]. The fatty acids in milk have positive effects on human health. For example, previous study showed that MUFAs had a positive effect on human health due to their cholesterol-lowering properties [29]. PUFAs are essential nutrients which play the important role in plasma lipids and endothelial function. Additionally, PUFAs can be also used in the prevention and treatment of coronary heart disease [30]. Hereby, we detected the potential SNP in the *SORBS*1 gene of cattleyak, and we revealed the effect of the SNPs in the *SORBS*1 CDS and their adjacent non-coding regions on the milk fat traits of cattleyak.

In this study, a total of nine SNPs located in the CDS and their adjacent non-coding regions of the *SORBS*1 gene in cattleyak were detected. After genotyping, all nine SNPs were found that have three genotypes in the population of cattleyak, and the dominant genotypes were mainly heterozygotes. In addition, our results showed that the genetic richness was high and had the potential of genetic variation, which lead to a more abundant selection effect. The natural selection, artificial selection intervention, genetic drift and other factors could lead to changes in some animal nucleotides so that the biological genetic traits become more colorful [31]. The size of the population genetic variation represents the level of the population genetic richness. Generally speaking, the higher the population genetic variation is, the higher is the population genetic richness. Thus, the PIC is often used as an indicator [32,33]. With the exception of g.29121 A > G, the other eight SNPs have reached the Hardy–Weinberg balance, which suggested that these SNPs of cattleyak were not strongly selected and might not be influenced by mutation, selection and genetic drift. It is also possible that the alleles and genotypes have a genetic advantage in their adaptability that rebalanced after a long period of artificial selection and breeding [34]. However, g.29121 A > G was out of balance, which means that traces of the artificial selection can be found. Thus, the other eight alleles are more likely to be rebalanced after a long period of artificial selection and reproduction.

In a previous study, it was generally assumed that only SNPs in exons were associated with biological traits, and these SNPs in introns did not affect biological traits [35,36,37]. In this study, five SNPs (g.29029 T > C, g.29050 A > G, g.29245 C > T, g.29305 C > T and g.29347 T > C), located in the introns of the *SORBS*1 gene were found to be significantly associated with the cattleyak milk fat traits. A recent study found that introns were involved in the gene splicing in eukaryotes, and that the mutation of introns might change the splicing efficiency, which resulted in the change of amino acid coding and eventually affected the biological traits [38,39]. Even if the intron mutation has no effect on the amino acid sequence, it may also result in the inactivation of the splicing site, or it may contain enhancer sequences to promote the specific transcription of the gene, affecting the animal phenotype [40,41]. The correlation between the SNPs on the introns and the milk fat traits of cattleyak may have a causal mutation, or it may be that these five SNPs are closely related to the SNPs on the exons. The exact cause needs further functional verification in a future study.

The effects of a specific gene on biological traits might be related to a combination of SNPs in that gene [42]. This study focuses on the genotype combination of SNPs causing amino acid changes. It was found that three SNPs (g.6256 C > T, g.24791 A > G, g.29121 A > G) caused amino acids changes and we obtained three haplotypes (Figure S1). Based on these three haplotypes, four combinations were obtained. The amino acids of the three SNPs in H1-H1 were changed compared with the standard sequence. Moreover, the milk fat content and fatty acid content of the H1-H1 population were significantly higher than those of other diplotypes. The milk fat content and fatty acid content of the H2-H2 population without amino acid mutation was the lowest. Therefore, it is clear that the mutation of amino acids has a positive effect on the percentage of milk fat and the content of fatty acids.

## 5. Conclusions

In conclusion, nine SNPs were detected in the CDS and their adjacent non-coding regions of the *SORBS*1 gene of the cattleyak, and all of these SNPs had a significant correlation with their milk fat traits. Additionally, H1-H1 (CCAAAA) is the dominant diplotype significantly related to the milk fat traits of cattleyak. These sites provide new molecular markers and a theoretical basis for the screening of the milk fat traits of cattleyak.

## Figures and Tables

**Table 1:** Conventional PCR amplification primers for CDS and their adjacent regions of the *SORBS*1 gene.

Primer Name	Sequence(5′-3′)	PCR Product Size (bp)	Region (bp)	Annealing Temperature (°C)
Primer 1	F: CACTTGCTCTCCCCTTCCTG R: CAACGTTCAGCCTCTGGACT	675	5791–6750	62
Primer 2	F: ATGCCCTGTGCTGTCAACTT R: TACAGTGGTCGCTGCCATAC	642	7741–8740	60
Primer 3	F: GGACAGGAGAGTTCTGTGGC R: AAGGACAGAGCTGCTGGAAC	939	18,181–19,180	63
Primer 4	F: AGAGTGCCTCACTGCATGTC R: ACAGACTGGTGAACAGCCAC	684	24,361–25,360	59
Primer 5	F: ACCGGATTGAGCCACAGTTT R: GGCACCAAGATTTTCCCAGC	789	28,681–29,680	60
Primer 6	F: ACTGAGGTCTCTCAGCCAGT R: TACAGTGGTCGCTGCCATAC	950	43,111–44,110	59
Primer 7	F: CTGTCTGACCCTGCTCTGTG R: GCCGGTGAGAAACTCAGGAA	680	79,641–80,640	61
Primer 8	F: TGCCATCTCCTCCCTACACA R: GTCCACACCATGGCCACTAA	721	85,681–86,680	61
Primer 9	F: CCAAGATGAGCACGGAAGGT R: GGGATTGTGGTGGTACCCAG	649	94,081–95,080	61
Primer 10	F: TCTCCAGACATCCCGTGTGA R: GGTCTTGTGGGCATCCACTT	952	96,061–97,060	61
Primer 11	F: GTTGAACGGATCTCCCCCAA R: GCAACTGGAAACTGCCCTTC	530	112,141–113,140	63
Primer 12	F: AAGCCCCTAACCTTGGTGTG R: AGAGCACGTGCAGGCTAAAT	790	114,001–115,000	61

Abbreviations: F = Forward primer; R = Reverse primer.

**Table 2:** SNP analysis of the population genetic parameters of *SORBS*1.

Loci	Genomic Region	Amino Acid	Genotype	Genotype Frequency (%)	Allele	Allele Frequency (%)	Ho	He	Ne	PIC	*p* Value
g.6256 C > T (c.298 C > T)	Exon 3	Ser100Pro	CC	38.3	C	61.9	0.528	0.472	1.894	0.361	0.054
			CT	47.1	T	38.1					
			TT	14.6							
g.24791 A > G (c.706 A > G)	Exon 7	Arg229His	AA	38.3	A	61.9	0.528	0.472	1.894	0.361	0.054
			AG	47.1	G	38.1					
			GG	14.6							
g.29029 T > C	Intron 7	-	TT	38.3	T	61.9	0.528	0.472	1.894	0.361	0.054
			TC	47.1	C	38.1					
			CC	14.6							
g.29050 A > G	Intron 7	-	AA	38.3	A	61.9	0.528	0.472	1.894	0.361	0.054
			AG	47.1	G	38.1					
			GG	14.6							
g.29121 A > G (c.979 A > G)	Exon 8	Ala327Thr	AA	48.0	A	66.7	0.556	0.444	1.799	0.346	0.017
			AG	37.4	G	33.3					
			GG	14.6							
g.29245 C > T	Intron 8	-	CC	40.6	C	63.0	0.534	0.466	1.873	0.358	0.073
			CT	44.8	T	37.0					
			TT	14.6							
g.29305 C > T	Intron 8	-	CC	40.6	C	63.0	0.534	0.466	1.873	0.358	0.073
			CT	44.8	T	37.0					
			TT	14.6							
g.29347 T > C	Intron 8	-	TT	40.6	T	63.0	0.534	0.466	1.873	0.358	0.073
			TC	44.8	C	37.0					
			CC	14.6							
g.96284 G > A (c.3090 G > A)	Exon 28	Ser1030Ser	GG	39.7	G	62.7	0.532	0.468	1.879	0.358	0.057
			GA	46.0	A	37.3					
			AA	14.3							

Abbreviations: Ho = Homozygosity; He = Heterozygosity; Ne = Effective number of alleles; PIC = Polymorphic information content. *p* > 0.05 suggested that the population gene is in Hardy–Weinberg balance and the sample comes from the same mendel population.

**Table 3:** Haplotype and haplotype frequencies of the three mutations in the *SORBS*1 gene.

Haplotype	Ploymorphism Sites of *SORBS*1 Gene			Frequency (%)
	g.6256 C > T (c.298 C > T)	g.24791 A > G (c.706 A > G)	g.29121 A > G (c.979 A > G)	
H1	C	A	A	61.8
H2	T	G	G	33.3
H3	T	G	A	4.9

**Table 4:** Association analysis between genotypes and the milk fat of cattleyak.

Loci	Genotype	Milk Fat Percentage (%)	MUFAs	PUFAs	SFAs
g.6256 C > T	CC	5.649 ± 0.499 ^a^	1.605 ± 0.212 ^a^	0.180 ± 0.018 ^a^	3.771 ± 0.388 ^a^
	CT	4.783 ± 0.732 ^b^	1.305 ± 0.177 ^b^	0.162 ± 0.023 ^b^	3.268 ± 0.479 ^b^
	TT	3.280 ± 0.677 ^c^	1.033 ± 0.231 ^c^	0.130 ± 0.011 ^c^	1.834 ± 0.529 ^c^
g.24791 A > G	AA	5.649 ± 0.499 ^a^	1.605 ± 0.212 ^a^	0.180 ± 0.018 ^a^	3.771 ± 0.388 ^a^
	AG	4.783 ± 0.732 ^b^	1.305 ± 0.177 ^b^	0.162 ± 0.023 ^b^	3.268 ± 0.479 ^b^
	GG	3.280 ± 0.677 ^c^	1.033 ± 0.231 ^c^	0.130 ± 0.011 ^c^	1.834 ± 0.529 ^c^
g.29029 T > C	TT	5.649 ± 0.499 ^a^	1.605 ± 0.212 ^a^	0.180 ± 0.018 ^a^	3.771 ± 0.388 ^a^
	TC	4.783 ± 0.732 ^b^	1.305 ± 0.177 ^b^	0.162 ± 0.023 ^b^	3.268 ± 0.479 ^b^
	CC	3.280 ± 0.677 ^c^	1.033 ± 0.231 ^c^	0.130 ± 0.011 ^c^	1.834 ± 0.529 ^c^
g.29050 A > G	AA	5.649 ± 0.499 ^a^	1.605 ± 0.212 ^a^	0.180 ± 0.018 ^a^	3.771 ± 0.388 ^a^
	AG	4.783 ± 0.732 ^b^	1.305 ± 0.177 ^b^	0.162 ± 0.023 ^b^	3.268 ± 0.479 ^b^
	GG	3.280 ± 0.677 ^c^	1.033 ± 0.231 ^c^	0.130 ± 0.011 ^c^	1.834 ± 0.529 ^c^
g.29121 A > G	AA	5.516 ± 0.621 ^a^	1.568 ± 0.225 ^a^	0.178 ± 0.021 ^a^	3.662 ± 0.516 ^a^
	AG	4.729 ± 0.715 ^b^	1.274 ± 0.152 ^b^	0.160 ± 0.020 ^b^	3.278 ± 0.401 ^b^
	GG	3.280 ± 0.677 ^c^	1.033 ± 0.231 ^c^	0.130 ± 0.011 ^c^	1.834 ± 0.529 ^c^
g.29245 C > T	CC	5.611 ± 0.588 ^a^	1.587 ± 0.231 ^a^	0.178 ± 0.021 ^a^	3.746 ± 0.465 ^a^
	CT	4.773 ± 0.696 ^b^	1.305 ± 0.167 ^b^	0.163 ± 0.022 ^a^	3.625 ± 0.428 ^b^
	TT	3.280 ± 0.677 ^c^	1.033 ± 0.231 ^c^	0.130 ± 0.011 ^c^	1.834 ± 0.529 ^c^
g.29305 C > T	CC	5.611 ± 0.588 ^a^	1.587 ± 0.231 ^a^	0.178 ± 0.021 ^a^	3.746 ± 0.465 ^a^
	CT	4.773 ± 0.696 ^b^	1.305 ± 0.167 ^b^	0.163 ± 0.022 ^a^	3.625 ± 0.428 ^b^
	TT	3.280 ± 0.677 ^c^	1.033 ± 0.231 ^c^	0.130 ± 0.011 ^c^	1.834 ± 0.529 ^c^
g.29347 T > C	TT	5.611 ± 0.588 ^a^	1.587 ± 0.231 ^a^	0.178 ± 0.021 ^a^	3.746 ± 0.465 ^a^
	TC	4.773 ± 0.696 ^b^	1.305 ± 0.167 ^b^	0.163 ± 0.022 ^a^	3.625 ± 0.428 ^b^
	CC	3.280 ± 0.677 ^c^	1.033 ± 0.231 ^c^	0.130 ± 0.011 ^c^	1.834 ± 0.529 ^c^
g.96284 G > A	GG	4.335 ± 0.772 ^c^	1.297 ± 0.377 ^b^	0.152 ± 0.029 ^b^	2.829 ± 0.679 ^b^
	GA	5.334 ± 0.612 ^a^	1.483 ± 0.167 ^a^	0.174 ± 0.021 ^a^	3.565 ± 0.467 ^a^
	AA	5.040 ± 0.283^b^	1.278 ± 0.088^b^	0.168 ± 0.011 ^a^	3.417 ± 0.024 ^a^

Abbreviations: MUFAs = Monounsaturated fatty acids; PUFAs = Polyunsaturated fatty acids; SFAs = Saturated fatty acids. ^a–c^ Values within a row with different superscripts differ significantly at *p* < 0.05.

**Table 5:** Association analysis of different genotype combinations of amino acid mutation sites with milk fat traits.

Diplotype	Combinatorial Genotype	Number of Amino Acid Mutations	Milk Fat Percentage (%)	MUFAs	PUFAs	SFAs
H1–H1	CCAAAA	3	5.649 ± 0.499 ^a^	1.605 ± 0.161 ^a^	0.180 ± 0.018 ^a^	3.771 ± 0.388 ^a^
H1–H2	CTAGAG	0–3	4.783 ± 0.595 ^b^	1.305 ± 0.143 ^c^	0.162 ± 0.020 ^c^	3.268 ± 0.396 ^b^
H1–H3	CTAGAA	1–3	4.992 ± 0.465 ^b^	1.421 ± 0.122 ^b^	0.171 ± 0.027 ^b^	3.321 ± 0.516 ^b^
H2–H2	TTGGGG	0	3.280 ± 0.652 ^c^	1.033 ± 0.153 ^d^	0.130 ± 0.011 ^d^	1.834 ± 0.262 ^c^

Abbreviations: MUFAs = Monounsaturated fatty acids; PUFAs = Polyunsaturated fatty acids; SFAs = Saturated fatty acids. ^a–d^ Values within a row with different superscripts differ significantly at *p* < 0.05.

## Data Availability

No new data were created or analyzed in this study. Data sharing is not applicable to this article.

## Supplementary Materials

The following are available online at www.mdpi.com/article/10.3390/ani11123461/s1. Figure S1. The mutation of SNPs caused the change of amino acid sequence (red maker).
