# Supplementary material for: Polymorphisms of SORBS1 Gene and Their Correlation with Milk Fat Traits of Cattleyak
Source: Animals (Basel). 2021 Dec 5;11(12):3461. doi: 10.3390/ani11123461 (PMC8697865; doi:10.3390/ani11123461)
Supplement: Supplementary file 1 [file animals-11-03461-s001.zip › animals-1467756-supplementary material (2).pdf]

Figure S1. The mutation of SNPs caused the change of amino acid sequence (red maker).

```
  1 MAYHSHYRDGETEALCGNDLSGVIRLVTAEATNPRRLVISILCCRKCDAG   50
    ||||||||||||||||||||||||||||||||||||||||||||||||||
  1 MAYHSHYRDGETEALCGNDLSGVIRLVTAEATNPRRLVISILCCRKCDAG   50

 51 ASKAVVNGLAPGSNGQDKATADPLRARSISAVKIIPVKTVKNSAGLVLPS  100
    |||||||||||||||||||||||||||||||||||||||||||||||||.
 51 ASKAVVNGLAPGSNGQDKATADPLRARSISAVKIIPVKTVKNSAGLVLPP  100

101 DMDPTRICTGKGAVTLRASSSYREIPSSSPVSPQETPKQERKTVLESENS  150
    ||||||||||||||||||||||||||||||||||||||||||||||||||
101 DMDPTRICTGKGAVTLRASSSYREIPSSSPVSPQETPKQERKTVLESENS  150

151 SADEWRLSSNADANGNAQPSSLAAKGYRSVHPSLPSSKPQDATSSSPAPP  200
    ||||||||||||||||||||||||||||||||||||||||||||||||||
151 SADEWRLSSNADANGNAQPSSLAAKGYRSVHPSLPSSKPQDATSSSPAPP  200

201 EVIVVPLYRVNTDRGHEGTDRPPASLGPRGPPVPAAAPAGSPLTFPTLDD  250
    ||||||||||||||||||||||||||||.|||||||||||||||||||||
201 EVIVVPLYRVNTDRGHEGTDRPPASLGPHGPPVPAAAPAGSPLTFPTLDD  250

251 FIPPHLQRRSHHSQPASAPGSLPPASQTPPSFSPQPPLVPPVPEGLRRVS  300
    ||||||||||||||||||||||||||||||||||||||||||||||||||
251 FIPPHLQRRSHHSQPASAPGSLPPASQTPPSFSPQPPLVPPVPEGLRRVS  300

301 EPDLTGAVSSTDSSPLLNEVSSSHAGADSQTFASVSKPSSAYPSTTIVNP  350
    ||||||||||||||||||||||||||.|||||||||||||||||||||||
301 EPDLTGAVSSTDSSPLLNEVSSSHAGTDSQTFASVSKPSSAYPSTTIVNP  350

351 TIVLLQHNREQQKRLSSLSDPVSERRAGEQDSAPAQEKPTSPGRAAERKA  400
    ||||||||||||||||||||||||||||||||||||||||||||||||||
351 TIVLLQHNREQQKRLSSLSDPVSERRAGEQDSAPAQEKPTSPGRAAERKA  400

401 KDDSRRVAKSAQDLSDVCMDEVGIPLRNTERSKDWYKTMFKQIHKLNRDT  450
    ||||||||||||||||||||||||||||||||||||||||||||||||||
401 KDDSRRVAKSAQDLSDVCMDEVGIPLRNTERSKDWYKTMFKQIHKLNRDT  450
```
